# Structuring polarization states of light in space and time

**DOI:** 10.1515/nanoph-2025-0438

**Published:** 2025-11-20

**Authors:** Danilo Gomes Pires, Jiaren Tan, Hooman Barati Sedeh, Natalia M. Litchinitser

**Affiliations:** Department of Electrical and Computer Engineering, 3065 Duke University, Durham, NC, USA

**Keywords:** spatiotemporal optical vortices, Poincaré sphere, optical skyrmions

## Abstract

The spatiotemporal sculpturing of light beams with arbitrary phase and polarization topologies has garnered significant attention in recent years due to its potential to advance optical technologies and reveal novel physical phenomena. Examples of spatiotemporal beams include space–time wave packets, flying donuts, tilted pulse fronts, X-waves, Airy pulses, and spatiotemporal optical vortices. Here, we introduce and demonstrate a new class of spatiotemporal polarization states of light. We propose a generalized spatiotemporal higher-order Poincaré sphere and show that these polarization states emerge from the superposition of two orthogonal circular polarization states, each carrying a spatiotemporal optical vortex. Such a choice of the basis enables simultaneous control of the spatial and temporal degrees of freedom of light. Theoretical predictions are experimentally validated using ultrafast femtosecond pulses, revealing how the resulting polarization distributions evolve in both space and time. Finally, we further extend this approach to construct a family of spatiotemporal skyrmionic textures that are localized, topologically nontrivial configurations of the electromagnetic field vector, offering a versatile framework for generating and controlling multidimensional (space and time) structured polarization fields. The ability to create and manipulate diverse forms of spatiotemporal skyrmionic textures opens up new opportunities for studying complex light–matter interaction phenomena, advanced imaging and micromanipulation, and encoding information across both space and time, with potential implications for advanced optical communication and information processing in classical and quantum domains.

## 1 Introduction

Conventional optics has traditionally emphasized plane waves and Gaussian beams; however, advances in singular optics and the orbital angular momentum (OAM) of light have introduced the concept of structured light. This development has expanded the capabilities of modern optics by enabling the generation and control of complex light fields with customized spatial and phase characteristics, opening new avenues for research and applications in imaging, communications, and quantum science. Spatially structured light beams include optical waveforms with spatial inhomogeneities in two or three dimensions, including beams with complex spin angular momentum (SAM) and OAM in both paraxial and non-paraxial regimes [1], [2], [3], [4], and vector beams with inhomogeneous polarization distributions [5], [6], [7]. Structured vector beams can be mathematically represented using the Poincaré sphere (PS) [8] and the higher-order Poincaré sphere (HOPS) [9], providing a geometric representation of vector beams composed of orthogonal spin–orbit states, such as superpositions of right (RCP) and left-circularly (LCP) polarized Laguerre–Gaussian (LG) modes carrying opposite OAM. These beams exhibit nonseparable coupling between spin and orbital degrees of freedom, leading to spatially varying polarization patterns such as radial, azimuthal, and hybrid vector fields. The HOPS framework has been essential for understanding the polarization topology of structured beams and has been extended to describe exotic light fields, finding applications in particle micromanipulation [10], [11], optical communications [12], [13], and sensing [14], [15].

Another class of vectorial beams, particularly interesting due to their topological properties in the spatial domain, is known as optical skyrmions. Skyrmions were first introduced in the context of condensed matter physics as topologically protected quasiparticles, possessing localized spin textures in which the magnetic moments continuously orient across space, forming stable, particle-like configurations [16], [17], [18], [19], [20], [21]. They have been observed in chiral magnets [22], [23], [24], and are well known for their resilience to external perturbations and potential for high-density, low-power information storage [25], [26], [27]. The idea of topological stability, based on the mapping between spatial coordinates and spin orientations in magnetic systems, has been extended to photonics through structured light. In optical skyrmions, the polarization or electric-field vector of light plays the role of the spin field, forming a similar mapping from the transverse spatial plane to the PS [28], [29], [30], [31], [32], [33], [34]. The resulting optical textures, such as Néel, Bloch, and anti-skyrmion textures, possess the same type of topological invariance. The skyrmion number quantifies how many times the optical vector field wraps around the Poincaré sphere and remains invariant under any continuous deformation of the field. This invariance provides optical skyrmions with intrinsic topological protection, ensuring that their global structure is preserved even in the presence of external perturbations [35], [36], [37], [38]. By extending the concept of topological order from condensed matter systems to free-space electromagnetic fields, optical skyrmions are likely to enable robust optical platforms for information processing and transmission.

In addition to spatial control, recent advances in temporal shaping of optical beams have introduced a new degree of freedom, enabling four-dimensional $\left(x,y,z,t\right)$ manipulation of light [39]. The combination of spatial and temporal beam shaping has given rise to spatiotemporal structured beams, such as spatiotemporal optical vortices (STOVs) [39], [40], [41], [42], [43], [44] that possess phase singularity in the joint space-time domain and an associated OAM carried by the pulse front rather than the wavefront. These beams exhibit nontrivial topological charges defined in the mixed transverse-temporal dimensions, leading to unique propagation dynamics such as transverse time shifts, tilted pulse fronts, and topologically protected energy flow. Moreover, in strong-field and nonlinear optics, STOVs provide novel ways to manipulate electron dynamics [45], [46], high-harmonic generation [47], [48], [49], [50], and filamentation processes [40], [51], revealing a new frontier in the topology of light that extends beyond purely spatial or temporal domains.

In this work, we introduce the spatiotemporal higher-order Poincaré sphere (STHOPS) as a novel framework for representing polarization dynamics jointly in space and time. This approach extends the concept of STOVs beyond scalar field distributions, enabling a unified description of spatial, temporal, and polarization degrees of freedom within a single representation. By modulating orthogonal vector functions across both space and time, we achieve continuous control over the polarization states on STHOPS, enabling the generation of a wide variety of spatiotemporal polarization vortices. While a spatio-spectral version of the Poincaré sphere was recently proposed to describe polarization across the frequency components of a pulse [52], our approach directly addresses the combined spatial and temporal domains. We further investigate how spectral transformations affect beam behavior, uncovering distinct polarization distributions in the spectral domain and unique polarization states in the temporal domain. Finally, we utilize this platform to create spatiotemporal optical skyrmionic textures, which represent topologically nontrivial polarization configurations that evolve in both space and time. These textures not only provide a new route for exploring fundamental aspects of light’s topology but also open exciting possibilities for various applications such as high-capacity information encoding, ultrafast optical communication, and advanced signal processing.

## 2 Theoretical model

The polarization states of light are commonly mapped into the well-known PS, where each unique point on its surface represents a particular state of polarization [8]. Within the spatial domain, the homogeneous polarization states of light can be described by a superposition of two orthogonal circular polarizations as

(1) $$\left|E_{\text{PS}}\right\rangle =\cos\left(\frac{\theta }{2}\right)\left|\sigma^{+}\right\rangle +\sin\left(\frac{\theta }{2}\right)e^{i\phi }\left|\sigma^{-}\right\rangle ,$$

where $\left|\sigma^{+}\right\rangle =\vec{x}+i\vec{y}$ and $\left|\sigma^{-}\right\rangle =\vec{x}-i\vec{y}$ correspond to the RCP and LCP states, and $\vec{x}$ and $\vec{y}$ are the orthogonal linear polarizations along the horizontal and vertical directions, respectively; the azimuthal (*ϕ*) and the polar (*θ*) angles determine the orientation and ellipticity of the polarization states, which can be retrieved in the experiment by measuring the Stokes parameters [53]. We note that while Eq. (1) enables the generation of different homogeneous polarization states along the PS, more complex, inhomogeneous polarization states can be achieved by linearly superposing two orthogonal circular polarizations carrying spatially structured amplitude and wavefront [9]. For instance, HOPS gives rise to a family of vectorial light fields, particularly those with spatially varying polarization. Taking as a basis the LG modes, which consist of an orthogonal set of solutions of the paraxial wave equation in cylindrical coordinates and can be written at the waist plane *z* = 0 in polar coordinates $\left(r,\varphi \right)$ as [4], [54]

(2) $$\text{L}{\text{G}}_{p,l}\left(r,\varphi \right)=\sqrt{\frac{p!}{\pi \left(\left|l\right|+p\right)!}}\frac{r^{\left|l\right|}}{w_{0}^{\left|l\right|+1}}{\text{L}}_{p}^{\left|l\right|}\left(\frac{r^{2}}{w_{0}^{2}}\right)e^{-\frac{r^{2}}{2w_{0}^{2}}}e^{il\varphi },$$

where the indices $\left(p,l\right)$ represent the radial and azimuthal indices, respectively, *w*_0_ is the beam waist, and ${\text{L}}_{p}^{\left|l\right|}$ is the generalized Laguerre polynomials with the radial index *p*, and azimuthal index *l*, which is known as topological charge and characterizes the phase singularity at the center of the beam. Now, Eq. (1) can be rewritten as [9]

(3) $$\left|E_{\text{HOPS}}\right\rangle =\cos\left(\frac{\theta }{2}\right)\text{L}{\text{G}}_{-l}\left|\sigma^{+}\right\rangle +\sin\left(\frac{\theta }{2}\right)\text{L}{\text{G}}_{l}e^{i\phi }\left|\sigma^{-}\right\rangle ,$$

where the optical modes LG_±*l*_ = LG_*p*=0,±*l*_ were introduced. Equation (3) describes the family of vectorial light states on the HOPS. Although Eq. (1) is defined as orthogonally polarized plane waves, we note that Eq. (3) reduces to the paraxial version of Eq. (1) when the constituent LG modes carry topological charge of *l* = 0.

**Figure 1:**
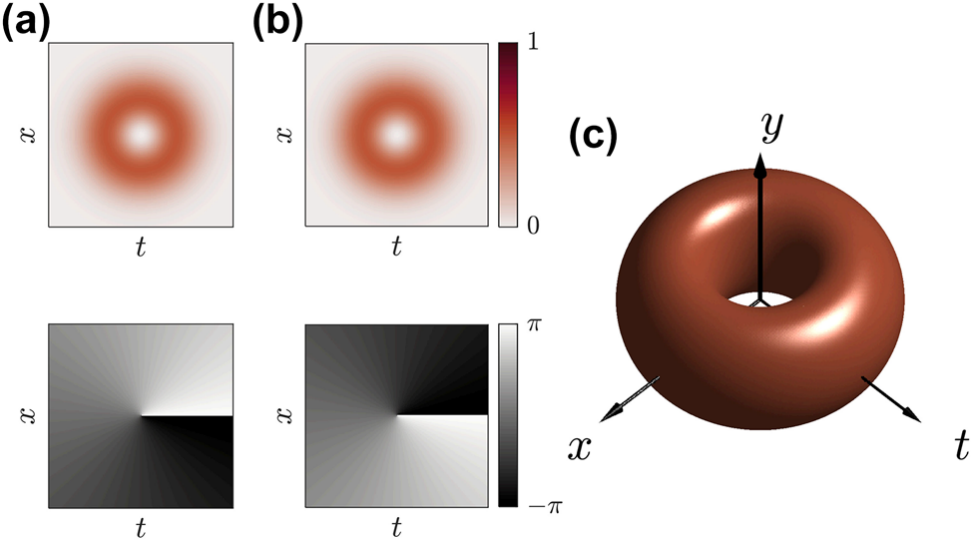
Respective intensity and phase distributions for a STOV carrying spatiotemporal topological charge of (a) *l* = −1 and (b) *l* = +1 from Eq. (5), which are used to create the STHOPS by superposing them within the spatiotemporal domain. (c) Iso-intensity distribution of a STOV in both space and time domains, highlighting the annular distribution across the $\left(x,t\right)$ plane and a Gaussian envelope along the *y*-direction.

Now, we introduce a method to construct an STHOPS by superposing two STOV states [41], [42] instead of the spatial-only LG beams. The spatiotemporal version of Eq. (3) is given by

(4) $$\begin{array}{ll} \left|E_{\text{STHOPS}}\right\rangle & =\cos\left(\frac{\theta }{2}\right)u{\left(x,y,z,t\right)}_{-l}\left|\sigma^{+}\right\rangle \\ & \;+\sin\left(\frac{\theta }{2}\right)u{\left(x,y,z,t\right)}_{l}e^{i\phi }\left|\sigma^{-}\right\rangle , \end{array}$$

where *θ*, *ϕ* denote the same polar and azimuthal angles as in the case of the original PS, and

(5) $$\begin{array}{ll} u{\left(x,y,z=0,t\right)}_{l} & =\sqrt{\frac{2}{{\left(\frac{x}{w_{0}}\right)}^{2}+{\left(\frac{t}{\tau }\right)}^{2}}}{\left(\frac{t}{\tau }+\mathrm{sgn}\left(l\right)\frac{ix}{w_{0}}\right)}^{\left|l\right|}\\ & \;e^{-\frac{x^{2}+y^{2}}{w_{0}^{2}}}e^{-\frac{t^{2}}{\tau^{2}}} \end{array}$$

stands for a *l*-th order STOV at *z* = 0, with *τ* denoting the pulse duration [41]. In contrast to the LG modes, STOVs possess a phase singularity line in the space-time domain, for example, in the direction perpendicular to the spatiotemporal plane $\left(x,t\right)$ , and a spatial Gaussian envelope along the *y*-direction. Notably, the first observation of these states was made during filamentation in air [40], [51], followed by several techniques developed to generate STOVs in laboratory settings, including the use of pulse shapers combined with holographic devices [55], [56], [57], [58] and optical metasurfaces [59], [60], [61]. Figure 1(a) and (b) shows the intensity and phase distributions of a −1 and +1 order STOV, respectively. An iso-intensity distribution is displayed in Figure 1(c) to illustrate the spatiotemporal profile of the STOV with order ±1

## 3 Experimental realization of STHOPS states

**Figure 2:**
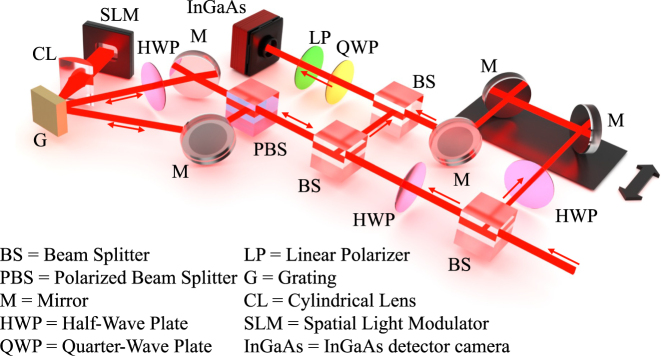
Experimental setup for measuring spatiotemporal polarization distributions, which combines Stokes polarimetry and a three-dimensional diagnostics technique. The pulse shaper is formed by a grating with 600/mm grooves, a cylindrical lens with *f* = 100 mm, and an SLM (Hamamatsu LCOS-SLM X10468-08). The data is acquired with a camera (Xenics Bobcat-320-GigE-8315), possessing an InGaAs detector sensor. For more details on Stokes polarimetry, refer to the Supplementary Materials.

Figure 2 shows an experimental setup developed to generate superposed spatiotemporal beams and perform a proper diagnostic within space, time, and polarization. A Ti: Sapphire pulsed laser with a pulse duration of 100fs and a repetition rate of 1 kHz passes through an optical parametric amplifier, generating near-infrared pulses centered at 1,550 nm. The initial beam is then separated using a 50/50 beam splitter (BS) into two routes, forming the signal and reference pulses. We rotate the polarization of the signal beam to 45° using a half-wave plate (HWP), followed by a polarized beam splitter (PBS) to further separate orthogonal linearly polarized pulses. Next, each of these orthogonally polarized pulses is redirected to the grating (G) and the output frequencies are collimated by a cylindrical lens (CL) before impinging on a spatial light modulator (SLM). Since the SLM only modulates light in one specific polarization state, an additional HWP has been introduced in one of the orthogonally polarized beams to ensure correct phase modulation. Spiral phase masks are used as phase-only holograms carrying opposite topological charges *l* = ±1 and were spatially separated within the SLM window. After the spatiospectral modulation, the pulses travel back and are combined after the PBS. The spatiotemporal vector beam, possessing opposite topological charges at RCP and LCP states, as described by Eq. (4), forms after the quarter-wave plate (QWP). The beam structure is retrieved by combining Stokes polarimetry and a three-dimensional diagnostic technique [55], [56]. The linear components of the Stokes parameters are measured by adding a linear polarizer (LP) immediately before the indium gallium arsenide (InGaAs) camera and rotating it by 0°, 90°, 45°, and 135°. Removing the linear polarizer allows us to measure the circular polarization components. The temporal profile of each spatiotemporal Stokes distribution is measured by scanning the modulated pulse with the reference beam, where the time delay between them is controlled by a delay stage (See Section 1 of the Supplementary Materials for the full description of the Stokes parameters). Note that the reference beam is configured to match the polarization state of the signal beam using an additional half-wave plate (HWP), which is essential for accurate interferometric measurements. The retrieved fields are then averaged over 10 measurements, with 100 temporal slices for each realization (For more details on the experimental averaging technique, see Section 2 of the Supplementary Materials).

**Figure 3:**
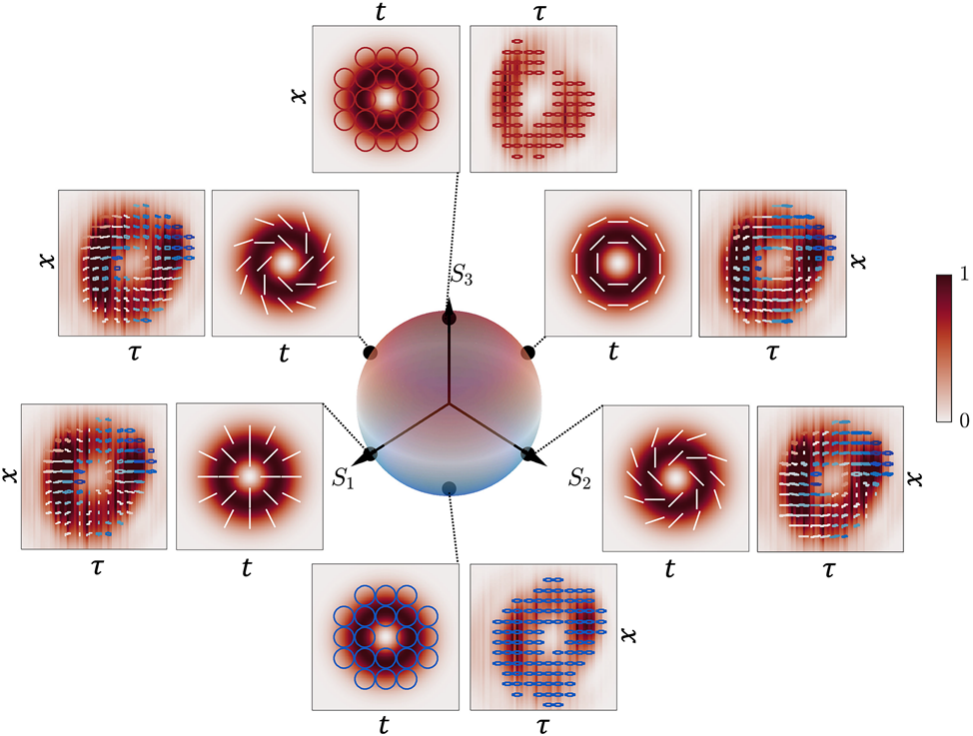
Normalized intensity and polarization distributions of the spatiotemporal vectorial states for the +1 order STHOPS. The North (South) pole corresponds to the RCP (LCP), and the states along the equator, *θ* = *π*/2, correspond to *ϕ* = 0, *π*/2, *π*, and 3*π*/2 with respect to *S*_1_. The experimental results are depicted with respect to the $\left(x,\tau \right)$ coordinates, where *τ* represents the time delay between the encoded and reference fields, while the theoretical distributions are shown with respect to the $\left(x,t\right)$ coordinates. The colored lines and circles represent the polarization state at that spatiotemporal region, where red (blue) represents the RCP (LCP) state and white represents linear polarization states at different angles. Here, the Stokes parameters are denoted by *S*_1_, *S*_2_, and *S*_3_.

**Figure 4:**
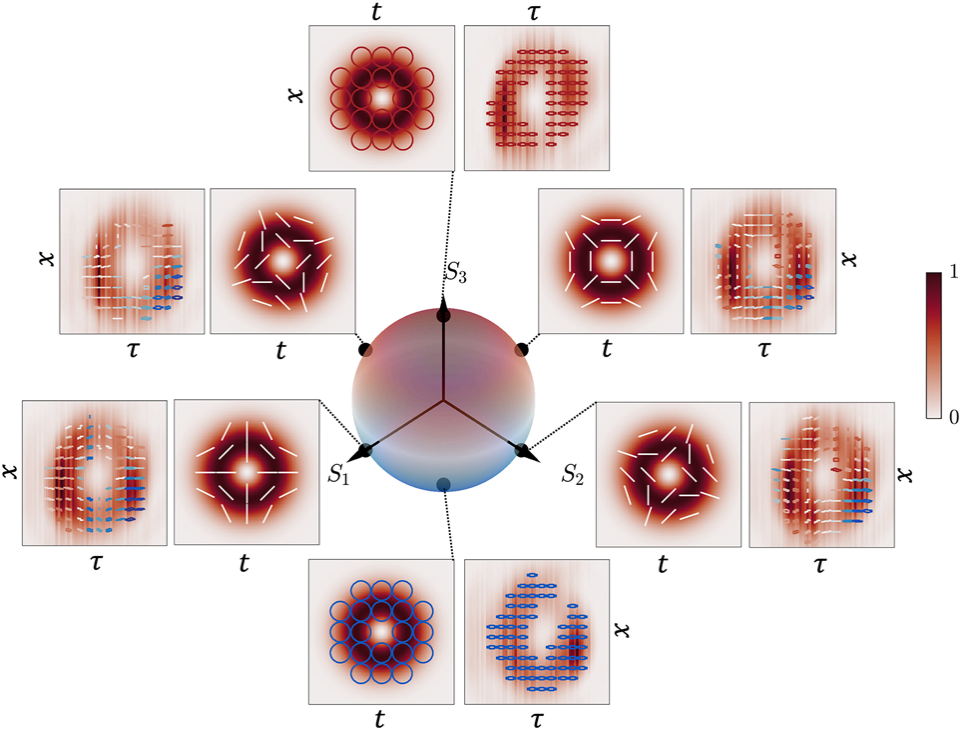
Normalized intensity and polarization distributions of the spatiotemporal vectorial states for the −1 order STHOPS. The North (South) pole corresponds to the RCP (LCP), and the states along the equator, *θ* = *π*/2, correspond to *ϕ* = 0, *π*/2, *π*, and 3*π*/2 with respect to *S*_1_. The experimental and simulation distributions are depicted in the same fashion as in Figure 3.

**Figure 5:**
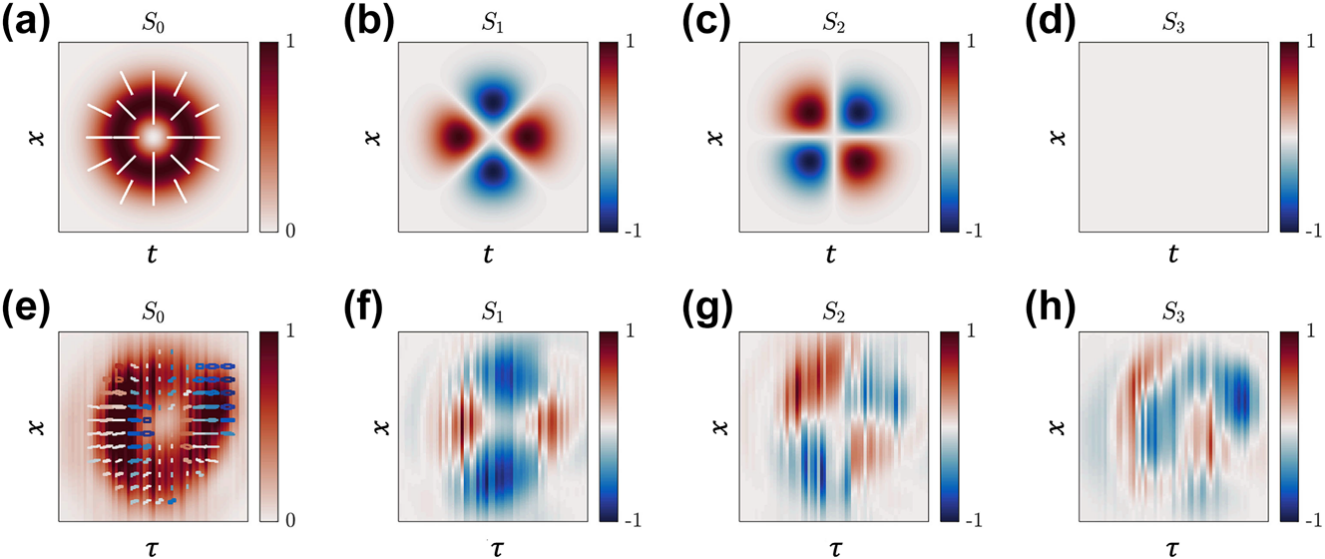
Stokes parameters $\left(S_{0},S_{1},S_{2},S_{3}\right)$ obtained from (a–d) theory and (e–h) experiments. Panels (a, e) show the theoretical and experimental intensity distribution together with their polarization states, respectively. Here, the colored lines and circles represent the polarization state at that spatiotemporal region, where red (blue) represents the RCP (LCP) state and white represents linear polarization states at different angles. Across all panels, the experimental results are depicted with respect to the $\left(x,\tau \right)$ coordinates, while the theoretical distributions are shown with respect to the $\left(x,t\right)$ coordinates.

The Stokes parameters *S*_1_, *S*_2_, *S*_3_ indicate the polarization difference between the horizontal and vertical $\left(S_{1}\right)$ , anti-diagonal and diagonal $\left(S_{2}\right)$ , and right-hand and left-hand circular $\left(S_{3}\right)$ states, respectively. In this context, the polarization difference between the states occurs within the spatiotemporal fields in Eq. (4), meaning that at each instant of time within the pulse, the beam possesses a different polarization distribution. In Figure 3, we show several examples of the states within the +1-order STHOPS, described by intensity and polarization distributions at multiple *θ*, *ϕ* angular locations along the +1-STHOPS surface, obtained from both theory and experiments. This is achieved by using Eq. (4) for the case of *l* = 1. Here, red circles denote RCP states $\left(\left|\sigma^{+}\right\rangle \right)$ and blue circles stand for LCP states $\left(\left|\sigma^{-}\right\rangle \right)$ . We note that by changing the topological charge to *l* = −1, we obtain another family of −1-order STHOPS, where several examples of the states are shown in Figure 4. It should be noted that while Eq. (4) describes a higher-order polarization state in the spatiotemporal plane, its spatio-spectral counterpart (*x*, *ω*) carries a different polarization distribution as it is outlined in Section 3 of the Supplementary Materials. An example of the Stokes parameters for the radially polarized state is shown in Figure 5. The theoretical (a-d) and experimental (e-h) Stokes parameters *S*_0_, *S*_1_, *S*_2_, *S*_3_ in Figure 5 correspond to the case of *l* = 1, *ϕ* = 0, and *θ* = *π*/2, along with their respective polarization distribution superposed in panels (a,e). Note that, due to the uneven intensity distribution in the experiments and the temporal mismatch of the superposing beams, some circular polarization leakage is observed. Whereas some quantitative differences remain, the polarization distributions obtained from the STHOPS measurements in Figures 3 and 4 capture the key trends predicted by theory, including the overall polarization rotation across the field. For instance, the spatiotemporal states located along the STHOPS equator show clockwise rotations for each local spatiotemporal polarization state along the ring-shaped intensity distribution matching the PS azimuthal coordinate for all *ϕ* = 0, *π*/2, *π*, 3*π*/2 states. This level of agreement supports the validity of the theoretical predictions while highlighting areas for further refinement in future experiments.

While the experimental results are generally consistent with theoretical predictions, and averaging techniques were applied to reduce post-processing effects (see Section 2 of the Supplementary Materials), some quantitative differences remain in the retrieved spatiotemporal polarization distributions. These deviations mainly arise from practical limitations in the experimental setup. Temporal synchronization and interferometric stability between the signal and reference arms are sensitive to mechanical drifts, which introduce complex field noise and slight temporal shifts during delay-line scans. Alignment imperfections in the pulse-shaping stage, particularly within the grating-cylindrical lens assembly, can cause small asymmetries in the reconstructed beam and polarization distributions. In addition, minor calibration errors in the Stokes polarimetry measurements may affect the relative scaling of the Stokes parameters, slightly influencing the extracted polarization angles and ellipticity. These factors explain the differences observed between theory and experiment and indicate that improved amplitude and phase control, active stabilization, and dispersion compensation could further enhance the accuracy of future spatiotemporal measurements.

## 4 Spatiotemporal skyrmionics textures

The construction of STHOPS establishes a unified formalism for describing light fields whose polarization is both spatially structured and intrinsically coupled to their temporal evolution. In this representation, each point on the sphere corresponds to a distinct non-separable polarization-time mode, extending the concept into the spatiotemporal domain. Building on the theoretical framework established in Section 2, the constituent fields in the superposition (Eq. (4)) can be chosen to generate more complex spatiotemporal structures, allowing access to a broader range of polarization and topologically structured light fields. Let’s now replace $u{\left(x,y,z,t\right)}_{-l}$ , describing −*l*-order STOV, by a Gaussian beam $G\left(x,y,t\right)=e^{-\frac{x^{2}+y^{2}}{w_{0}^{2}}}e^{-\frac{t^{2}}{\tau^{2}}}$ , while keeping $u{\left(x,y,z,t\right)}_{l}$ in the second term of Eq.(4) unchanged. This construction is given by

(6) $$\begin{array}{ll} \left|E_{\text{S}}\right\rangle & =\cos\left(\frac{\theta }{2}\right)G\left(x,y,t\right)\left|\sigma^{+}\right\rangle +\sin\left(\frac{\theta }{2}\right)\\ & \;u{\left(x,y,z,t\right)}_{l}e^{i\phi }\left|\sigma^{-}\right\rangle , \end{array}$$

results in a formation of skyrmionic beams, which arise as topologically protected textures of the electromagnetic field, where the polarization vectors (or equivalently the local Stokes vectors) wrap the sphere in a nontrivial manner [28], [30], [34], [35], [57]. While these textures have been widely explored in the spatial domain, few studies have considered the realization of optical skyrmions confined in space and time [57], [62], [63], [64], [65]. Here, we experimentally observe the analogous spatiotemporal skyrmionic textures that are realized by appropriately tailoring the superposed fields and extending the concept of optical skyrmions into the joint space-time domain. These structures exhibit polarization vortices and domain-wall-like features in combined space-time coordinates, enriching the accessible topological phase space.

Among the wide variety of skyrmionic textures, Néel, Bloch, and Anti-type skyrmions represent distinct topological configurations of vector fields, originally classified in magnetic systems but now extended to optical, acoustic, and other wave domains [22], [23], [24], [25], [26], [27], [28], [29], [30], [31], [32], [33], [34], [35], [36], [37], [57], [62], [64]. In a Néel-type skyrmion, the field vectors rotate radially, pointing inward or outward from the skyrmion core, leading to a hedgehog-like texture where the helicity angle is 0 or *π*. In contrast, a Bloch-type skyrmion exhibits azimuthal rotation of the field vectors around the core, leading to a vortex-like structure with helicity ± *π*/2. These two canonical types differ by the sense of rotation in the local field orientation, reflecting different forms of chiral symmetry breaking. An Anti-skyrmion, on the other hand, possesses a mixed topology in which the rotation direction varies along orthogonal axes, giving rise to anisotropic textures with alternating helicity signs and negative skyrmion number. Despite their structural differences, all skyrmion types share the fundamental property of topological protection. This means that their continuous field configuration cannot be transformed into a trivial state with no singularities or discontinuities, ensuring robustness against perturbations such as disorder, noise, or turbulence [30], [35], [36], [37], [38]. As previously discussed, in optics, the different types of skyrmions, including Néel, Bloch, and anti-skyrmions, appear as structured polarization and phase distributions in which the local Stokes parameters define a continuous mapping of the optical field onto the PS.

**Figure 6:**
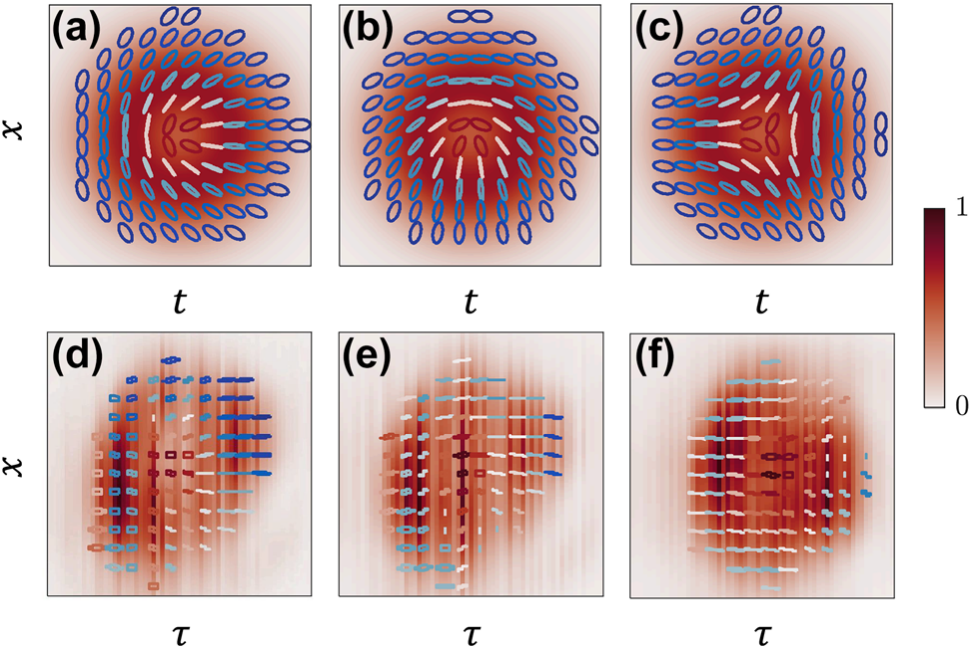
Spatiotemporal skyrmionic textures constructed from (a–c) theory and (d–f) experiments. Here, we present the (a, d) Néel, (b, e) Bloch, and (c, f) Anti-skyrmion textures. The polarization states are represented by colored lines and circles, where red (blue) stands for the RCP (LCP) state and white stands for linear polarization states at different angles. Similar to Figures 3, 4, and 5, the experimental results are depicted with respect to the $\left(x,\tau \right)$ coordinates, while the theoretical distributions are shown with respect to the $\left(x,t\right)$ coordinates.

Examples of the theoretical and experimental spatiotemporal textures corresponding to Néel-type optical skyrmions are shown in Figure 6(a) and (d), respectively, using *l* = 1, *θ* = *π*/2, *ϕ* = *π*. The Bloch-type spatiotemporal optical skyrmions are also shown in Figure 6(b) and (e), with *l* = 1, *θ* = *π*/2, *ϕ* = 3*π*/2, respectively. Finally, the anti-skyrmion-type spatiotemporal textures are shown in Figure 6(c) and (f) by using *l* = −1, *θ* = *π*/2, *ϕ* = *π*, respectively. The skyrmion numbers of the respective skyrmionic textures observed experimentally in Figure 6 (e) and (f) are calculated with respect to the reduced Stokes parameters $s=\left(s_{1},s_{2},s_{3}\right)$ through [57]

(7) $$N=\frac{1}{4\pi }\iint s\cdot \left(\frac{\partial s}{\partial x}\times \frac{\partial s}{\partial \tau }\right)\text{d}x\text{d}\tau ,$$

where each reduced Stokes parameters are written as *s*_*i*_ = *S*_*i*_/*S*_0_ and the integration is performed across the spatiotemporal cross-section, defined in the experiment as the spatial axis *x* and the time delay between the signal and reference pulses *τ*. The skyrmion number is 1 for Néel- and Bloch-type skyrmions, and −1 for Anti-type skyrmions. The skyrmion numbers obtained from the experiments are found to be 0.95 for the Néel-type texture, 0.99 for the Bloch-type texture, and −0.90 for the anti-skyrmion-type texture. In addition to the skyrmion number measurements, the experimental textures show similar local polarization states and overall distributions to the numerical results, with polarization evolving in the same direction across the field. For example, the Néel-type skyrmions in Figure 6(d) exhibit polarization rotation along the azimuthal direction that agrees well with the theoretical pattern shown in Figure 6(a). In the Bloch-type textures shown in Figure 6(b) and (e), a helicity rotation of approximately −*π*/2 relative to the Néel case is observed, with the local polarization rotating clockwise along the azimuthal direction. Finally, the Anti-type skyrmion exhibits the expected reversal of polarization orientation on opposite sides of the beam center. This can be visualized by following the polarization rotation along a line passing through the center of the field distribution. These skyrmionic textures may open new opportunities for data storage, optical communication, sensing, and metrology.

In our experiments, for both STHOPS and spatiotemporal skyrmionic textures, the results were obtained using a phase-only hologram of the phase singularity in the $\left(x,\omega \right)$ plane, rather than a full amplitude and phase modulation of the STOVs across the spatiotemporal domain. This technique is commonly used to generate optical vortices carrying orbital angular momentum without any radial orders at the far-field and was widely used to do so in both space-only [59], [60], [61] and spatiotemporal domains [41], [42], [55]. To validate this approach, Section 4 of the Supplementary Materials demonstrates that employing a phase-only hologram, as in the experimental arrangement, yields equivalent results.

## 5 Conclusions

In summary, we have introduced a new class of spatiotemporal polarization states of light arising from the coherent superposition of spatiotemporal optical vortices carrying opposite topological charges in orthogonal circular polarization states. This approach establishes the STHOPS as a generalized framework that unifies the spatial, temporal, and polarization degrees of freedom of light within a single representation. The theoretical predictions, validated experimentally through full spatiotemporal Stokes polarimetry, reveal controllable transitions across the STHOPS surface. By tuning the relative phase and amplitude of the constituent modes, we realized a variety of spatiotemporal polarization structures, extending the known family of vector beams into the joint space-time domain.

Experimentally, the shaping of the optical pulse in space, time, and polarization was achieved through a compact and flexible interferometric arrangement incorporating basic optical components and an SLM. By encoding phase-only holograms carrying opposite topological charges on each orthogonally polarized component, we were able to impose independent modulations along both spatial and spectral dimensions. This method enables direct and reconfigurable control over the pulse’s spatiotemporal structure, allowing the generation of vector beams with arbitrary polarization evolution in time. The combination of holographic modulation, polarization optics, and interferometric synchronization thus provides a robust platform for constructing high-dimensional optical states without requiring complex or multi-stage modulation schemes.

Building upon this foundation, we further constructed spatiotemporal optical skyrmionic textures, representing localized and topologically protected configurations of the electromagnetic field that evolve coherently in space and time. The experimentally measured skyrmion numbers confirm the realization of Néel-, Bloch-, and Anti-type spatiotemporal skyrmions, marking an extension of multiple optical skyrmionic textures into the spatiotemporal regime. These results highlight the potential of the STHOPS framework as a universal platform for generating, visualizing, and manipulating complex light fields with nontrivial topology.

Beyond their fundamental significance, the demonstrated spatiotemporal polarization states open new directions for exploring nonseparable light–matter interactions, ultrafast topological photonics, and multidimensional information encoding. Their ability to couple space, time, and polarization within a single optical entity offers new routes for structured-field control, high-capacity optical communication, and quantum information processing. Altogether, this work provides a comprehensive foundation for the emerging field of spatiotemporal structured light and establishes new opportunities for advancing both classical and quantum photonic technologies.

While this work focused on the STHOPS-based approach for polarization dynamics in the (*x*, *t*) plane, future research will focus on extending this study to higher-dimensional spatiotemporal vector fields. A recent report [66] indicates that topological textures spanning four-dimensional domains can be realized by combining structured light with strong focusing under a microscope objective or by integrating temporal light modulation with metasurfaces or liquid-crystal phase retarders. The experimental platform developed here is well-suited for such studies, as it enables simultaneous control of spatial, temporal, and polarization degrees of freedom.

## References

[1] Andrews D. L., Babiker M. (2012). *The Angular Momentum of Light*.

[2] Marrucci L. (2011). Spin-to-orbital conversion of the angular momentum of light and its classical and quantum applications. *J. Opt.*.

[3] Allen L., Padgett M., Babiker M. (1999). IV The orbital angular momentum of light. *Progress in optics*.

[4] Yao A. M., Padgett M. J. (2011). Orbital angular momentum: Origins, behavior and applications. *Adv. Opt. Photon.*.

[5] Zhan Q. (2009). Cylindrical vector beams: From mathematical concepts to applications. *Adv. Opt. Photon.*.

[6] Maurer C. (2007). Tailoring of arbitrary optical vector beams. *New J. Phys.*.

[7] Ndagano B. (2016). Beam quality measure for vector beams. *Opt. Lett.*.

[8] Poincare H. (1954). Theorie mathematique de la lumiere (Gauthiers-Villars, Paris 1892) Vol. 2. *J. Opt. Soc. Am.*.

[9] Milione G. (2011). Higher-order Poincaré sphere, Stokes parameters, and the angular momentum of light. *Phys. Rev. Lett.*.

[10] Xu F., Wang L., Mu R., Zheng F., Jiang M., Wang G. (2024). Levitated 2D manipulation on dielectric metasurface by the tuning of polarization states. *Opt. Lett.*.

[11] Ha G. (2019). Polarization effect on optical manipulation in a three-beam optical lattice. *Phys. Rev. A*.

[12] Milione G. (2015). Using the nonseparability of vector beams to encode information for optical communication. *Opt. Lett.*.

[13] Zhao Y., Wang J. (2015). High-base vector beam encoding/decoding for visible-light communications. *Opt. Lett.*.

[14] Wu H. (2021). Cylindrical vector beam for vector magnetic field sensing based on magnetic fluid. *IEEE Photon. Technol. Lett.*.

[15] Yu W., Yan J. (2024). Modelling and analysis of vector and vector vortex beams reflection for optical sensing. *Photonics*.

[16] Perring J., Skyrme T. (1962). A model unified field equation. *Nucl. Phys.*.

[17] Skyrme T. (1958). A non-linear theory of strong interactions. *Proc. Roy. Soc. Lond. Ser. A. Math. Phys. Sci.*.

[18] Skyrme T. (1959). A unified model of K-and π-mesons. *Proc. Roy. Soc. Lond. Ser. A. Math. Phys. Sci.*.

[19] Skyrme T. (1961). Particle states of a quantized meson field. *Proc. Roy. Soc. Lond. Ser. A. Math. Phys. Sci.*.

[20] Skyrme T. H. R. (1961). A non-linear field theory. *Proc. Roy. Soc. Lond. Ser. A. Math. Phys. Sci.*.

[21] Skyrme T. H. R. (1962). A unified field theory of mesons and baryons. *Nucl. Phys.*.

[22] Muhlbauer S. (2009). Skyrmion lattice in a chiral magnet. *Science*.

[23] Han J. H. (2010). Skyrmion lattice in a two-dimensional chiral magnet. *Phys. Rev. B Condens. Matter Mater. Phys.*.

[24] Yu X. (2018). Transformation between meron and skyrmion topological spin textures in a chiral magnet. *Nature*.

[25] Yu G. (2017). Room-temperature skyrmion shift device for memory application. *Nano Letters*.

[26] Luo S., You L. (2021). Skyrmion devices for memory and logic applications. *APL Mater.*.

[27] Zhang X. (2015). Skyrmion-skyrmion and skyrmion-edge repulsions in skyrmion-based racetrack memory. *Sci. Rep.*.

[28] Shen Y. (2024). Optical skyrmions and other topological quasiparticles of light. *Nat. Photonics*.

[29] Tamura R. (2024). Direct imprint of optical skyrmions in azopolymers as photoinduced relief structures. *APL Photon.*.

[30] Yang A. (2025). Optical skyrmions: From fundamentals to applications. *J. Opt.*.

[31] Tsesses S. (2018). Optical skyrmion lattice in evanescent electromagnetic fields. *Science*.

[32] Shen Y., Martínez E. C., Rosales-Guzmán C. (2022). Generation of optical skyrmions with tunable topological textures. *Acs Photonics*.

[33] McWilliam A. (2023). Topological approach of characterizing optical skyrmions and multi‐skyrmions. *Laser Photon. Rev.*.

[34] Gao S. (2020). Paraxial skyrmionic beams. *Phys. Rev. A*.

[35] Wang A. A. (2024). Topological protection of optical skyrmions through complex media. *Light: Sci. Appl.*.

[36] Zhang Z. (2025). Topological protection degrees of optical skyrmions and their electrical control. *Photon. Res.*.

[37] Nagaosa N., Tokura Y. (2013). Topological properties and dynamics of magnetic skyrmions. *Nat. Nanotechnol.*.

[38] Nape I., Singh K., Klug A. (2022). Revealing the invariance of vectorial structured light in complex media. *Nat. Photon.*.

[39] Shen Y. (2023). Roadmap on spatiotemporal light fields. *J. Opt.*.

[40] Jhajj N. (2016). Spatiotemporal optical vortices. *Phys. Rev. X*.

[41] Hancock S. (2019). Free-space propagation of spatiotemporal optical vortices. *Optica*.

[42] Chong A. (2020). Generation of spatiotemporal optical vortices with controllable transverse orbital angular momentum. *Nat. Photon.*.

[43] Bliokh K. Y. (2021). Spatiotemporal vortex pulses: Angular momenta and spin-orbit interaction. *Phys. Rev. Lett.*.

[44] Zhang H. (2023). Topologically crafted spatiotemporal vortices in acoustics. *Nat. Commun.*.

[45] Chen Y. (2023). Atomic photoionization by spatiotemporal optical vortex pulses. *Phys. Rev. A*.

[46] Sun F. (2025). Isolated attosecond γ-ray pulse generation with transverse orbital angular momentum using intense spatiotemporal optical vortex lasers. *Phys. Rev. Appl.*.

[47] Chen Z.-Y. (2022). Relativistic high-order harmonic generation of spatiotemporal optical vortices. *Phys. Rev. A*.

[48] Gui G. (2021). Second-harmonic generation and the conservation of spatiotemporal orbital angular momentum of light. *Nat. Photon.*.

[49] Hancock S. W., Zahedpour S., Milchberg H. M. (2021). Second-harmonic generation of spatiotemporal optical vortices and conservation of orbital angular momentum. *Optica*.

[50] Martín-Hernández R. (2025). Extreme-ultraviolet spatiotemporal vortices via high harmonic generation. *Nat. Photon.*.

[51] Le M. (2024). Self-focused pulse propagation is mediated by spatiotemporal optical vortices. *Phys. Rev. Lett.*.

[52] Fickler R., Kopf L., Ornigotti M. (2024). Higher-order Poincaré spheres and spatiospectral Poincaré beams. *Phys. Rev. Res.*.

[53] Berry H. G., Gabrielse G., Livingston A. (1977). Measurement of the Stokes parameters of light. *Appl. Opt.*.

[54] Allen L., Beijersbergen M. W., Spreeuw R. J. C., Woerdman J. P. (1992). Orbital angular momentum of light and the transformation of Laguerre-Gaussian laser modes. *Phys. Rev. A*.

[55] Cao Q. (2022). Non-spreading Bessel spatiotemporal optical vortices. *Sci. Bull.*.

[56] Chen W. (2025). Tailoring spatiotemporal wavepackets via two-dimensional space-time duality. *Nat. Commun.*.

[57] Teng H. (2025). Construction of optical spatiotemporal skyrmions. *Light: Sci. Appl.*.

[58] Mounaix M. (2020). Time-reversed optical waves by arbitrary vector spatiotemporal field generation. *Nat. Commun.*.

[59] Turnbull G. A. (1996). The generation of free-space Laguerre-Gaussian modes at millimetre-wave frequencies by use of a spiral phaseplate. *Opt. Commun.*.

[60] Shalaev M. I. (2015). High-efficiency all-dielectric metasurfaces for ultracompact beam manipulation in transmission mode. *Nano Lett.*.

[61] Rosales-Guzmán C., Forbes A. (2017). *How to Shape Light with Spatial Light Modulators*.

[62] Vo S., Gutiérrez-Cuevas R., Alonso M. (2024). Closed forms for spatiotemporal optical vortices and sagittal skyrmionic pulses. *J. Opt.*.

[63] Bliokh K. Y., Nori F. (2012). Spatiotemporal vortex beams and angular momentum. *Phys. Rev. A– At., Mol., Opt. Phys.*.

[64] Smirnova D. A., Nori F., Bliokh K. Y. (2024). Water-wave vortices and skyrmions. *Phys. Rev. Lett.*.

[65] Wang B. (2025). Topological water-wave structures manipulating particles. *Nature*.

[66] Marco D., Alonso M. A. (2025). 4D topological textures in light. *Phys. Rev. Lett.*.

